# Swedish adaptation of the General Medical Council's multisource feedback questionnaires: a qualitative study

**DOI:** 10.5116/ijme.5af6.c209

**Published:** 2018-06-15

**Authors:** Jan-Eric Olsson, Solvig Ekblad, Bo Christer Bertilson, Eva Toth-Pal

**Affiliations:** 1 Academic Primary Healthcare Centre, Stockholm County Council, Stockholm, Sweden; 2 Department of Learning, Informatics, Management and Ethics, Cultural Medicine, Karolinska Institutet, Stockholm, Sweden

**Keywords:** Multisource feedback questionnaires, translating, qualitative research, education, medical, graduate, self- assessment

## Abstract

**Objectives:**

to explore potential users’ opinions of a translated and culturally adapted Swedish version of the General Medical Council's MultiSource Feedback Questionnaires.

**Methods:**

In this qualitative study, we used content analysis on semi-structured interviews from 44 resident doctors, 29 medical colleagues and 28 patients to analyse their opinions of the Swedish adapted version, created through translation and expert review. Transcribed interview data concerning the informants’ general thoughts about the tool were coded manually by three independent coders into categories, compiled as themes, and exemplified by citations. Data regarding specific question wording and relevance were used as a basis for final questionnaire revision.

**Results:**

The informants valued the tool’s potential to provide essential feedback to support the development of residents' medical competences and communication skills. Resident doctors welcomed support in their self-reflection. Colleagues saw it as a valuable tool for assessment that needs to be used sensitively. Patients appreciated opportunities to communicate feedback.  Ambiguous or irrelevant questions and response options were identified. Some colleague-related questions about specific skills and knowledge appeared ambiguous to residents. The final questionnaire revision - based on the expert review and the interview analysis - resulted in a number of changes: four questions were deleted, twelve were reformulated, and six were added.

**Conclusions:**

Potential users perceived the Swedish adapted version as a beneficial tool for residents in their professional development. Further research is needed to explore how this tool can influence doctors’ development when used in real-life settings.

## Introduction

Resident doctors need evidence-based clinical tools to obtain comprehensive feedback on their clinical performance during their specialist training to inform their professional development.

In order to attain a better alignment between the syllabus and professional milestones, the mission statement for resident doctors in family medicine in Sweden was revised in 2015.^1^ Formative, Work Place Based Assessment (WPBA) methods^2^ were identified as important for contributing information about doctor’s ways of acting - described as the “does” level according to the Miller framework.^3^

One validated 360-degree assessment tool for assessing doctors' clinical skills on the "does" level, is MultiSource Feedback (MSF), originally developed for use in medical education by Van der Vleuten.^4^ MSF is one of the WPBA methods and involves inviting different groups of assessors to answer questionnaires that ask about their experience of residents’ work over an extended period.^4^ Assessor groups can consist of supervisors, peers, other colleagues, or patients. Using multiple assessor groups increases the validity of the results and reduces bias. Patients’ contributions mainly reflect aspects of empathy, professionalism and communication skills experienced in occasional meetings with the resident doctors.^5^^,^^6^ There is good scientific evidence that MSF is a powerful method for achieving behavioural change among professionals,^7^ and MSF tools have been developed and used in many English-speaking countries.^8^^,^^9^ The impact of MSF ratings from different sources has, however, also been under question.^10^ Critics argue that assessors’ loyalties to colleagues, and the sampling method of the assessors, might weaken the credibility of MSF as a tool for identifying poor performance.^11^ On the other hand, Bullock and colleagues, found that nurses and consultants were significantly more critical in their ratings than junior doctors.^12^  MSF is still one of the better tools to assess interpersonal communication, professionalism, and teamwork behaviours according to Donnon and colleagues.^13^^,^^7^

A literature review performed by the first author during 2014 showed that the General Medical Council Multisource Feedback Questionnaires (hereafter referred to as the GMC Questionnaires) from the UK meet high demands for documentation, comprehensive validation, psychometric tests, and extensive use.^14^

The GMC Questionnaires consist of three tools – one to be filled in by the assessed doctor (self-assessment) (SQ), one by their colleagues (CQ), and one by their patients (PQ). Some sections of questions are common in the three questionnaires, which gives an opportunity to compare the results. The core focus of the original GMC Questionnaires is good medical praxis in clinical work of doctors. The GMC Questionnaires are recommended and accepted as a tool and play an important part in the revalidation process of doctors in the UK since 2012.^15^^,^^16^

Until now validated WPBA tools to improve the specialist training for doctors have not been deployed in Sweden, resulting in demand by healthcare authorities and by directors of studies for their introduction. To fill this gap, we translated the GMC Questionnaires and adapted it to the Swedish context. In this study, we explored potential users’ opinions of the adapted Swedish version of the GMC Questionnaires, while the authors have described the psychometric properties of the tool in a separate article.^17^

## Method

### Study design

This is a qualitative study following consolidated criteria for reporting qualitative research (COREQ) recommended by Tong and colleagues.^18^ We used qualitative content analysis,^19^^-^^22^ on verbatim transcribed semi-structured interviews with residents, colleagues, and patients to describe their opinions of the translated and culturally adapted GMC Questionnaires. Residents examined SQ; colleagues examined CQ, and patients PQ. In the content analysis, we followed an interview guide (Table 1) created for this purpose. The informants were asked to give their general thoughts about the questionnaires, their perceptions of the relevance of specific questions and their interpretation of the wordings.

### Procedure

There are no consensus guidelines for cross-cultural adaptation of questionnaires.^23^^,^^24^ We performed translations, back-translation and expert review in our cross-cultural adaptation of the GMC Questionnaires.^25^ The English questionnaires are not copyrighted and are freely available.  Two independent professional translators did the translations and back-translation. The translated version was revised by three expert groups and two bilingual doctors together with the research team. The expert groups consisted of directors of studies in family medicine, senior paediatric physicians and representatives of the Swedish Medical Association.  This process resulted in the revised Swedish GMC version tested in this study.  Questions about people's race and ethnic group are prohibited in Sweden by law and were removed from the background information in the SQ. Some adjustments of questions were made to better fit within the context of Swedish residents in family medicine. Eight new questions were introduced including questions about the patient-centred approach, language skills, and demographic information (See Appendix).

**Table 1:** The semi-structured interview guide for all interviews

The interview guide	
After the informants have had time to read through the form undisturbed for some time, we asked the following general question:	
1.	When you have read all the questions in the questionnaire, what are your thoughts about it? [Continue with the specification of comments on each question in the tool]:
2.	What does this question mean to you?
3.	What do you think of when you read this question?
4.	What do you think about the wording of this question? Is it clear enough so you can answer it unambiguously?
5.	Do you have any other comments?

### Participants

During the period 19 February to 11 June 2015, employees at four medical centres, including groups of resident doctors, colleagues and patients in Stockholm and the surrounding area were asked by email or face to face to participate in the interviews either in focus groups or individually. Our goal was to recruit 20–30 informants from each target group (residents, colleagues and patients) to reach demographic representativeness. Informants were recruited through convenience sampling. A total of 101 informants participated in 16 focus group interviews and 29 individual interviews (Table 2). The informants consisted of 44 resident doctors, 29 medical colleagues representing several professions (17 doctors, 12 co-workers including nurses, secretaries, laboratory staff, and assistant nurses) and 28 patients. Sixty-two (61%) of the 101 informants in the interviews were female. Patients were between 18 and 85 years of age, and half of them were younger than 50 years. None of the recruited informants dropped out.

Ethical approval was obtained from the Regional Ethical Review Board in Stockholm on December 4, 2014. Participation in the interviews was entirely voluntary and possible to cancel at any time. Informants were given oral and written information and signed informed consent. To ensure anonymity, all the results including citations were presented so that no informant could be identified.

**Table 2:** Characteristic of informants in the interviews (N = 101)

Informant group	Female n (%)	Male n (%)	All	focus groups n	individual interviews n
Residents	26 (59)	18 (41)	44	9	1
Colleagues	18 (62)	11 (38)	29	6	0
Patients	18 (64)	10 (36)	28	1	24
Total	62 (61)	39 (39)	101	16	25

### Data collection and analysis

Data were collected in 16 focus group- and 25 individual interviews. The interviews took part at different clinics and workplaces and lasted between 35 and 66 minutes (average 55 minutes). A moderator and an observer led each focus group interview. The moderators were two males and five females; either experienced general practitioners and directors of studies in family medicine or licensed psychologists. All authors took part in the interviews as moderators or observers. The moderators took part in a common training session before the interviews. The observers wrote field notes and reported back to the informants at the end of each session to check whether the information was correctly understood. The interviews were electronically recorded and transcribed verbatim, and then coded manually by three members of the research team. All content was marked for the identification of codes. The codes were grouped into categories and exemplified by citations. Coding of each transcribed interview text was done by two members of the research team independently. Codes and categories were then compared and discussed until consensus was reached. The whole research team then discussed the compiled categories and themes. Finally, three themes developed, one for each group of informants (residents, colleagues and patients) elucidating the latent content. Data regarding specific question wording and relevance were used as a basis for final questionnaire revision.

## Results

All informants perceived the Swedish version of the GMC tool as containing relevant questions and as reflecting the most important perspectives in the medical profession. They valued the tool’s potential to provide essential feedback from patients and colleagues to support the development of residents' medical competences and communication skills. The three themes and associated categories for each group of informants are summarised in Table 3.

### Residents' theme 

#### Feedback from different perspectives stimulate self-reflection and development

The residents valued the comprehensive nature of the tool whereby others' assessments provide support for development and self-reflection about medical skills and communication.  The analysis identified six categories, and we include some relevant citations to illustrate the content of the categories:

#### A new and useful tool describing important medical competences

The residents perceived that the content was relevant and comprehensive. The questions were in line with the description of the objectives for their specialty and addressed essential aspects of how a doctor should work. Questions on clinical knowledge, decision making, and consultation skills were perceived to be the most important issues.

“It covers quite a lot of what you are expected to know and be good at as a doctor... it seems quite comprehensive” Transcript 16, resident, male

“Well yes, it seems as though they [the questions] are quite strongly rooted in the description of our objectives” Transcript 7, resident, female

#### The tool can provide essential support for assessment and development

Residents perceived that this tool could be a support for their development by pointing out areas for improvement as well as areas where they were insecure or had missed during previous training. They pointed out the importance that their supervisors had access to the results of the tool so that they could use it as a basis for common discussions and follow-ups. They suggested that this tool could be a complement to other assessment methods during the residency period.

“I think we must have that [the tool] quite simply. Otherwise we won’t improve.” Transcript 12, resident, male

” You can quite easily see… in which areas you are, like, a little less sure about.”  Transcript 9, resident, female

#### A tool for self-reflection

Residents reported that this tool could help them reflect on their work and development. The possibility to compare the results

from the self-assessment questionnaire with the assessments from patients and colleagues was perceived to give important information for how to work towards further development.

“And it’s rewarding, I can reflect on myself. Like, how have I worked? How have things turned out, for example?” Transcript 10, resident, female

“I believe that it’s extremely important to receive feedback about how to develop and to receive it from colleagues and the patient at the same time, then for me it’s like looking in the mirror” Transcript 6, resident, male

**Table 3:** Themes and categories concerning opinions from residents, colleagues and patients

Residents' theme: feedback from different perspectives stimulate self-reflection and development
Categories:
· A new and useful tool describing important medical competences
· The tool can provide essential support in assessment and development
· A tool for self-reflection
· Different colleagues can assess different competencies
· Patients' assessments are particularly important
· Communication is an important competence where language skills matter
Colleagues' theme: the tool assesses important competencies and needs to be used sensitively
Categories:
· The tool addresses important issues
· It might feel uncomfortable to be rated, but also rewarding
· Different professionals' perspectives contribute differently to the assessment
· Language skills are important and relevant
Patients' theme: a relevant tool to express patients’ views
Categories:
· Well-formulated, well-structured, and relevant questions
· An important tool for patients where they can communicate both negative and positive views
· The questionnaire contains questions that reflect the patient's needs in a medical encounter
· The doctor's language skills are important for communicating with the patient

#### Different colleagues can assess different competencies

The residents felt that there are competencies in the medical profession that not all colleagues can assess. According to the residents, you must know the doctor well and see the doctor working closely with patients to make a complete assessment of what happens behind the doctor's closed door.

“You have to know the colleague quite well and to have worked with them for quite some time.” Transcript 12, resident, male

“It has to be people who have been in the room… you’re quite alone in your room so few people know how you actually work” Transcript14, resident, female

#### Patients' assessments are particularly important

The residents expressed that patients' participation and feedback were particularly valuable and essential for them because these could provide a more comprehensive picture of their skills and how well they perform their work.

“I think the best thing is to approach the patients and ask them… It would be good to hear it from the patients… They’re who you work for… most important is the patient’s evaluation” Transcript 5, resident, male

#### Communication is an important competence where language skills matter

Residents perceived that a doctor’s communicative skills are of crucial importance in contact with patients, relatives, and colleagues. To be able to communicate verbally and in writing was seen as an essential part of a doctor’s work.

“Communication with the patient is extremely important. If… there are like shortcomings in the language, then both the doctor and the patient can misunderstand the situation. Language is a big part of the interaction with the patient.” Transcript 10, resident, female

### Colleagues' theme

#### The tool assesses important competencies and needs to be used sensitively

Colleagues, including doctors, nurses, and other coworkers, reported that this is a useful and powerful tool that should be used considerately, as feedback assessment can be a delicate matter. We found four categories:

#### The tool addresses important issues

Colleagues experienced that the tool addressed important and relevant questions of how a doctor works. They perceived that the most important issues were medical knowledge, consultation skills, a patient-centred approach, achieving trust, and recognising and working within limitations.

“I think they’re good points. They’re relevant points for assessing how a doctor works well." Transcript 12, colleague, female

“if you give this to the right person, it can result in very important and beneficial feedback." Transcript 12, colleague, male

“Consultation skills… being able to communicate with patients that are as important as knowledge …” Transcript 1, colleague, male

It might feel uncomfortable to be rated, but also rewarding

To be assessed by colleagues and patients might feel uncomfortable at first, but also rewarding in the long run. Colleagues perceived that the GMC Questionnaires provided a well-structured and comprehensive assessment that the residents had to endure in order to develop and improve.

“I thought that maybe it’s difficult to hear, but then it’s probably very rewarding as well.”  Transcript 3, colleague, male

“I feel a bit sorry for them because it’s a very detailed assessment of everything… Although I guess it’s about them improving themselves.” Transcript 2, colleague, female

#### Different professionals' perspectives contribute differently to the assessment

Colleagues appreciated that the GMC Questionnaires created a combined assessment from many employees because different professionals might have difficulties rating all questions.

“How should I assess whether the residents… strive for continuity in the patient relation.” Transcript1, colleague, male

 “…your assessment is one of maybe four or five… at the moment it’s just the supervisor who assesses most often, and the vice-principal, whether the residents are good in practice.” Transcript 1, colleague, male

#### Language skills are important and relevant

Because of a large number of immigrant doctors in Sweden, their language skills are a very relevant question and a part of the communication that is very important to make the patient feel secure. According to the colleagues, it is a problem when a patient does not understand what the doctor is saying.

“It’s very difficult to work as a doctor if others can’t understand what you’re saying, so it’s a relevant question.” Transcript 3, colleague, female

“How you express yourself to the patient. Because it feels, many of them feel that… they’re not satisfied with the visit because of the language… communication is extremely important, and many lack that aspect.” Transcript 8, colleague, female

### Patients’ theme

#### A relevant tool to express patients´ views

Patients perceived the tool as a well-formulated feedback form describing relevant parts of the patient-doctor encounter.  We identified the following three categories: 

#### Well-formulated, well-structured, and relevant questions

The patients appreciated the focus on the patient and that the doctor´s mission was clear. They perceived that the aspects of knowledge and communication were important.

“There were relevant questions, I think, about interaction… but also about knowledge.” Transcript 20, patient, female

“It becomes clear who they [the doctor] are there for.” Transcript 20, patient, female

#### An important tool for patients where they can communicate both negative and positive views

The patients appreciated the questionnaire as a follow-up of the doctor-patient encounter where they could express both negative and positive views. Patients can often feel dependent in the encounter and feel that it is a delicate situation to criticize a doctor. They welcomed a tool that documents both strengths and weaknesses in the doctor's competencies.

“I myself can sometimes feel that when you’ve been to the doctor that… you have quite a lot of opinions about sometimes but nowhere to express them…” Transcript 18, patient, female

“You feel very small, you’re always at a disadvantage as a patient and even more so if you have a complicated health issue.” Transcript 29, patient, female

#### The questionnaire contains questions that reflect the patient's needs in a medical encounter

Patients perceived that the questions relate to exactly what a patient would wish to ask to be satisfied with a doctor-patient encounter. One important issue was that the doctor could correctly assess the patient's condition.

“It’s exactly this [what the questionnaire addresses] that should … occur during a, a doctor’s appointment… and if it occurs during a doctor’s appointment you’re completely happy.” Transcript 19, patient, female

“Yes, it’s very important that the doctor can assess my medical condition and help me get treatment.” Transcript 33, patient, female

#### The doctor's language skills are important for communicating with the patient

Being able to communicate in Swedish was perceived by the patients as a relevant aspect in the questionnaire. Patients expressed that they felt it was crucially important to understand what the doctor means, especially when being in an exposed situation.

“I think the question as to whether the doctor understands or speaks Swedish is highly relevant… when you’re lying there in a vulnerable situation in a hospital. So you’re extremely anxious to truly understand everything that is happening and being discussed.” Transcript 17, patient, male

#### Comments on wording and relevance of specific questions

Comments on specific questions were the largest part of the interviews, and there were comments on the wording and relevance of two thirds of the questions. Colleague-related questions about specific skills and knowledge were perceived as being more difficult to interpret than questions in the patient-related section. Especially the resident informants perceived that the colleague-related questions needed to be clearer and easier to answer.

The comments on wording and relevance of specific questions were sorted into four groups – ambiguous questions, ambiguous response options, irrelevant questions, and irrelevant response options. Examples of the most common issues for each group are presented below (see Appendix).

#### Ambiguous questions in SQ/CQ

The interpretation of the question “Recognizing and working within limitations” in the Swedish version was difficult for all residents and five of the six focus groups with colleagues. We found at least five different interpretations, which led us to reformulate the question to “Recognizing and working within own limitations”. One third of the focus groups were doubtful about the question “Working effectively with colleagues”. They considered the word “effectively” to be inappropriate and their suggestion was “Cooperation with colleagues”

#### Ambiguous response options in SQ/CQ

The Swedish word for “poor” in the answer options to the colleague-related questions evoked negative reactions in five groups. It was perceived as being too emotive, and one informant reported that the term is often avoided in the Swedish education context.

#### Irrelevant questions in PQ/SQ

The contextual patient question about the importance of the visit was controversial because some patients felt that all visits were important for them. The question whether the doctor respected patient confidentiality, was considered too complicated to be answered by four of the resident groups as well as by many patients.

#### Irrelevant response options (CQ)

Response options “more than five years ago” – in the question “How recently have you been familiar with this doctor’s clinical practice” was considered inappropriate because both residents and colleagues thought that it would be too difficult to remember a colleague's performance after such a long time. A few colleagues were concerned that the demographic/background information options in questions 6–8 might reveal their identity.

### Final version

A number of changes were made to the three questionnaires based on the suggestions from the expert groups and the comments regarding specific question wording and relevance in the interviews. The answer options in the 5-point scale of the colleague-related questions were changed to insufficient, not satisfactory, satisfactory, good, and very good (back-translated from Swedish). Differences between the final Swedish version and the original GMC Questionnaires are presented in the Appendix.

## Discussion

The revised Swedish version of the GMC Questionnaires was well received by potential users as residents, colleagues, and patients who participated in the interviews. However, the informants had many comments on the wordings and relevance of specific questions that influenced the final version. All informants in the three target groups expressed positive expectations on the use of the tool regarding its ability to support residents' development in their clinical skills. The introduction of new questions concerning language skills and patient-centeredness were also considered relevant by all three groups of informants. Residents, colleagues, and patients also had their themes and specific opinions concerning the benefits, strengths, and weaknesses of the tool. The residents appreciated the opportunity for self-reflection and welcomed the tool as a new tool for supporting their professional development. On the other hand, they questioned whether all of their colleagues had enough insight into the work of the resident to make a valid rating. The same discussion occurred in the interviews with colleagues, and some of them expressed that they would not be able to answer all of the questions. The colleagues´ theme: “The tool assesses important competencies and needs to be used sensitively” expressed that it could be unpleasant, but also rewarding to be assessed and that it depended on the assessors' profession as to which aspects of the resident's work they could assess. The positions of doctors and patients in the medical encounter were described as unequal, where patients felt dependent on the doctor and therefore might be hesitant in revealing their emotional, and rational reactions face to face with the doctor. They appreciated this tool because it focused on the patient's perspective and provided new opportunities for anonymous feedback. Also, the residents expressed that the patients' assessments were especially crucial for them.

Even though many reflections on wording appeared in the interviews, not all of them led to changes in the final revision because we were keen to preserve the primary structure of the questionnaires so that they would be comparable with the original. Some of the individual colleague and patient-related questions were ambiguous or irrelevant according to many of the informants and were thus reformulated or removed by the research team in the final version. The question whether "this doctor respects patient confidentiality" was too ambiguous to use in our context and was erased. In the Swedish context, this question can implicate two scenarios – to keep personal information about patients confidential or to obey the prohibition against reading patient records from other clinics without the patients’ consent. The latter injunction provokes some strong reactions amongst clinicians who see it as compromising the quality of patient care. We had to reformulate the response options in the 5-point scale of the colleague-related questions because there were many objections to the wording when translated to Swedish. We based the reformulated wording on the terminology of the European Credit Transfer and Accumulation System, ECTS,^26^ because it is already in use at several Swedish universities. Suggestions from residents to use numbers instead of words in the answer options were rejected because it would deviate too much from the original concept of the GMC Questionnaires.

There are several ways to translate and adapt a questionnaire from one language to another, and adaptation and validation are two different processes that should be distinguished.^24^ We, therefore, chose to perform these processes in two different studies.  We considered qualitative content analysis^27^^,^^28^ as a useful method to explore the informants’ perceptions of the whole questionnaire and to simultaneously analyse semantic, idiomatic, experiential, and conceptual interpretations of individual questions in comparison with the originals.^29^^,^^20^ In our previous psychometric validation study,^17^ the translated and adapted Swedish version was analysed for internal consistency and construct validity by calculating Cronbach’s alpha and performing Principal Component Analysis and Confirmatory Factor Analysis. The results confirmed the tool as being a reliable and valid tool.

In a qualitative follow-up study of the GMC Questionnaires by Hill and colleagues informants were concerned whether patients and colleagues can provide objective feedback.^11^ Archer and colleagues saw the risk of combining scores from different sources and leniency bias to be validity problems in the MSF method.^10^ Also, in some of our interviews, some colleagues reflected on the trustworthiness of ratings from colleagues and the effect of leniency bias, especially regarding ratings on language skills. But the majority of our informants did not express such apprehension.

As described in the literature, it depends on the professional group which questions they can answer in an MSF tool.^30^ Depending on the profession, colleagues can observe the resident in different professional situations. This is also confirmed both by our previous validation study and by the results of this study.^17^ Colleagues in our interviews considered it to be scary as well as rewarding to be assessed. However, none of them had participated in an MSF assessment, so their opinions describe only their expectations. This concern was supported by the Hill and colleagues study above, where some participating informants actually were upset by their scores.^11^ Feelings of distress were also found in follow-up interviews from those who got feedback lower than their self-perceptions in a small Canadian study of another MSF tool from 2006.^31^ Negative feedback might thus cause resistance to the MSF method if not properly handled,^32^ and careful considerations are required, especially when using MSF as a summative assessment. The Swedish versions of the GMC Questionnaires are planned to be used in formative assessments during the residence period with the aim to find weaknesses in the residents' performance and to give opportunities to correct them.

In other WPBA tools like mini-CEX and mini-PAT, assessors are requested to compare their ratings with the expected level of the doctors’ competence.^2^ We tried to introduce such a comparison for the colleague-related questions, but without success. This was because both the residents and colleagues reported that they did not know the expected levels in sufficient detail to be able to make this comparison.

### Limitations

The main limitation of this study, regarding possibilities to transform the results to an anticipated user satisfaction of the tool is that the informants’ opinions in the interviews reflected only their first impression of the Swedish GMC Questionnaires and was not based on actual participation in the assessment process. This was, though, not avoidable as the Swedish version of the tool was new. A possible weakness was also that the first author, who was observer or moderator in some focus group interviews, was known to many of the residents, which might have created a leniency effect on their opinions about the tool. However, we found no evidence for this in our analysis of the interviews. Citations are only identified by group and gender, but not by age and not always by profession. This was because in some interview groups with mixed professions it was not always possible to discern different voices.

### Implications of the study

Access to this tool opens new possibilities for residents to improve the quality of their clinical work and professional skill development, and this may benefit patients. There is now an online version of the Swedish GMC Questionnaires, including the possibility of online delivery of the feedback results, which makes this tool available on a larger scale. Further education of tutors and colleagues is needed about how to use this tool for feedback and how to build a constructive dialogue with the residents to create an open and feedback-friendly climate in the workplace that is necessary for an optimal result. The Swedish version of the GMC tool may be a valuable resource in combination with other forms of formative assessment in professional development.  There is also the potential to use the tool in other clinical specialities and at different levels of medical education.

## Conclusions

We found in this study that potential users perceived the adapted Swedish GMC Questionnaires as a beneficial tool for residents in their professional development. Further research is needed to explore how this tool can influence the medical education of future professionals on different levels in their development of clinical skills when used in real-life settings.

### Acknowledgements

The authors acknowledge our project administrator Anneli Lagerqvist for her dedicated effort to transcribe 617 pages from the tape-recorded interviews; the irreplaceable contributions of the patients, colleagues, and residents who participated in the interviews; Ed Peile, Emeritus Professor in Medical Education at the University of Warwick who gave us the project idea and proofread the manuscript; and Helena Schildt-Tossman, MD, head of the vocational training department of the Academic Primary Health Centre for her support throughout the entire project.

### Conflict of Interest

The authors declare that they have no conflict of interest.

## Supplementary material

Supplementary file 1 Appendix. Differences between the final Swedish version and the original GMC Questionnaires
