# Supplementary material for: Swedish adaptation of the General Medical Council's multisource feedback questionnaires: a qualitative study
Source: Int J Med Educ. 2018 Jun 15;9:161–9. doi: 10.5116/ijme.5af6.c209 (PMC6129165; doi:10.5116/ijme.5af6.c209)
Supplement: Supplementary file 1 — Appendix. Differences between the final Swedish version and the original GMC Questionnaires [file ijme-9-161-S1.pdf]

Appendix

Differences between the final Swedish version and the original GMC Questionnaires concerning new, removed, and reformulated questions in the Self-evaluation Questionnaire (SQ), the Colleague Questionnaire (CQ), and the Patient Questionnaire (PQ)

Changes made during this research	Questions translated from Swedish	SQ	CQ	PQ
New questions introduced by the expert team	Gives the patient the opportunity to talk about her/his anxiety and fears	X		X
	Patient-centred approach	X	X	
	Attaching importance to continuity in patient relationships	X	X	
	This doctor has difficulty expressing himself/herself in Swedish in speech or writing	X	X	X
	This doctor has difficulty in understanding Swedish	X	X	X
	Was there an interpreter at the consultation?			X
Questions removed by the expert team	This doctor's performance is not impaired by ill health	X	X	
	Respects patient confidentiality*	X	X	X
	Colleague's ethnic group	X		
	Patient's ethnic group	X		
	Was the patient's visit with their usual doctor?			X
Reformulated questions by the expert team and based on the interviews	Gives the patient a good reception	X		X
	Makes the patient feel safe	X		X
	Diagnostic thinking	X	X	
	Recognizing and working within own limitations	X	X	
	Cooperation with colleagues	X	X	
	I think that this doctor can give me good care			X
	I will gladly meet with this doctor again			X
	Suitable to take care of patients		X	X
	Gender			X
	Age group			X
	Colleague's profession			X
	Provides constructive, specific, and comprehensible feedback on strengths and weaknesses in the box for narrative "free text" comments	X	X	X

*Question removed based on the interviews in the final revision
